# Andrographolide Activates Keap1/Nrf2/ARE/HO-1 Pathway in HT22 Cells and Suppresses Microglial Activation by A*β*_42_ through Nrf2-Related Inflammatory Response

**DOI:** 10.1155/2017/5906189

**Published:** 2017-03-08

**Authors:** Ji Yeon Seo, Euisun Pyo, Jin-Pyo An, Jinwoong Kim, Sang Hyun Sung, Won Keun Oh

**Affiliations:** Korea Bioactive Natural Material Bank, Research Institute of Pharmaceutical Sciences, College of Pharmacy, Seoul National University, Seoul 08826, Republic of Korea

## Abstract

Therapeutic approach of Alzheimer's disease (AD) has been gradually diversified. We examined the therapeutic and preventive potential of andrographolide, which is a lactone diterpenoid from* Andrographis paniculata*, and focused on the Kelch-like ECH-associated protein 1 (Keap1)/nuclear factor (erythroid-derived 2)-like 2 (Nrf2)-mediated heme oxygenase (HO)-1-inducing effects and the inhibitory activity of amyloid beta (A*β*)_42_-induced microglial activation related to Nrf2 and nuclear factor *κ*B (NF-*κ*B)-mediated inflammatory responses. Andrographolide induced the expression and translocation of Nrf2 from the cytoplasm to the nucleus, thereby activating antioxidant response element (ARE) gene transcription and HO-1 expression in murine hippocampal HT22 cells. Andrographolide eliminated intracellular A*β*_42_ in BV-2 cells and decreased the production of interleukin (IL)-6, IL-1*β*, prostaglandin (PG)E_2_, and nitric oxide (NO) because of artificial phagocytic A*β*_42_. It decreased pNF-*κ*B accumulation in the nucleus and the expression of inducible nitric oxide synthase (i-NOS) and cyclooxygenase II (COX-II) in the microglial BV-2 cell line. In summary, andrographolide activates Nrf2-mediated HO-1 expression and inhibits A*β*_42_-overexpressed microglial BV-2 cell activation. These results suggested that andrographolide might have the potential for further examination of the therapeutics of AD.

## 1. Introduction

Therapeutic approaches for brain diseases, including Alzheimer's and Parkinson's diseases, amnesia, and ischemia, were extensively studied in the aging era [1]. Neuroinflammation is one of the main alterations that occur in brain disease, and the characteristics of neuroinflammation are accompanied by the induction of cytokines, as IL-1*β* and 6. Microglial activation, which is one of the pathological features of neuroinflammation, can also be accompanied by amyloid *β* plaque formation and hyperphosphorylated and misassembled microtubule-associated tau proteins and their aggregates in the brains of Alzheimer's patients [2, 3]. These pathological features are observed depending on the AD stages and they produce and release multiple types of inflammatory mediators or cytokines through microglial activation by several stimulators such as A*β* in aged microglia. On the other hand, several studies have reported that A*β*-related peptides which are large secreted N-terminal portion of the protein (sAPP), adhesion associated amyloid precursor protein (APP), multimerization associated APP, A*β* oligomer, and A*β* fibrils [4] could initiate microglial activation. These microglia can eliminate the myelin or cell debris as well as it can also assist A*β* clearance through phagocytic removal [5]. The phytochemicals help these roles of microglia; for example, some studies have reported the activities of curcumin [6] and resveratrol [7] on A*β* clearance in microglia or their effects on elimination of A*β* peptides. Therefore, it is meaningful to search for candidate clearing A*β* oligomers or plaques that can be used in anti-Alzheimer drug discovery. Commonly, Nrf2, a well-known transcription factor, acts as a powerful regulator that induces not only detoxifying enzymes, such as nicotinamide adenine dinucleotide phosphate (NADPH) oxidoreductase 1, glutathione S-transferase in the liver, gamma-glutamyl cysteine synthase, and HO-1 [8], but also anti-inflammatory mechanisms associated with NF-*κ*B-mediated inflammatory responses accompanying i-NOS, COX-II enzyme expression, cytokine release, and NO production [9]. HO-1 is a strong antioxidant and detoxifying enzyme that exerts cytoprotection in brain [10]. The study about Nrf2 as a regulator protein of NF-*κ*B has recently been reported; thereby the underlying mechanism of natural compounds related to Nrf2 and Keap1 also has not been fully understood so far. 

Andrographolide, which is a lactone diterpenoid, is present in the medicinal plant* Andrographis paniculata*. This plant has been used as a functional food or for medicinal purposes in several human diseases in Asia and Europe [11]. HMPL-004, a standardized extract of the plant* Andrographis paniculata* containing a high concentration of andrographolide, was clinically evaluated in patients with mild to moderate ulcerative colitis (UC). Double-blind and placebo-controlled clinical phase 2 trials of HMPL-004 reported that this plant preparation at a daily dose of 1,800 mg generated a positive response in terms of its safety and effectiveness compared with those receiving placebo. This research guarantees the safety and developmental possibility of andrographolide [12]. The activity of andrographolide has also been reported, and its therapeutic roles in the regulation of inflammation in microglia [13] have been described.* In vivo* studies examining the therapeutic aspects of andrographolide have shown that* Andrographis paniculata* extract and andrographolide ameliorated the memory deficits not only induced in the diabetes model of rats [14] but also induced in APPswe/presenilin-1 (PS-1) AD model of mice [15]. In addition, it is also associated with the stimulation of neurogenesis in hippocampus [16].

Although the effects of andrographolide have been associated with the Nrf2 signaling pathway in several cell types, such as human hepatoma cells and the NF-*κ*B-mediated inflammatory signaling pathway induced by lipopolysaccharide in RAW264.7 macrophages and murine microglia BV-2 cells [13], its role in neurons and microglial activation remains unknown. Thus, we investigated whether andrographolide induces Nrf2-mediated HO-1 activity in immortalized mouse hippocampal cells and NF-*κ*B-mediated inflammation inhibitory activities during microglial activation induced by A*β*_42_ in microglial BV-2 cells through Nrf2 action.

## 2. Materials and Methods

### 2.1. Cells

The immortalized mouse hippocampal HT22 cell line was obtained from Pro. Jong-Sang Kim's laboratory and Prof. Dong-Seok Lee's laboratory at Kyungpook National University. The murine microglial BV-2 cell line was obtained from Korea Research Institute of Bioscience and Biotechnology. Both cell lines were grown in Dulbecco Modified Eagle Medium (DMEM)/high-glucose medium supplemented with 10% fetal bovine serum (FBS) and 100 U/mL of penicillin and 100 *μ*g/mL of streptomycin (P/S). HT22 cells were passaged every two or three days at a 1 to 3–8 ratio, and BV-2 cells were subcultured at a 1 to 2-3 ratio every day.

### 2.2. Chemicals and Reagents

**Figure 1:**
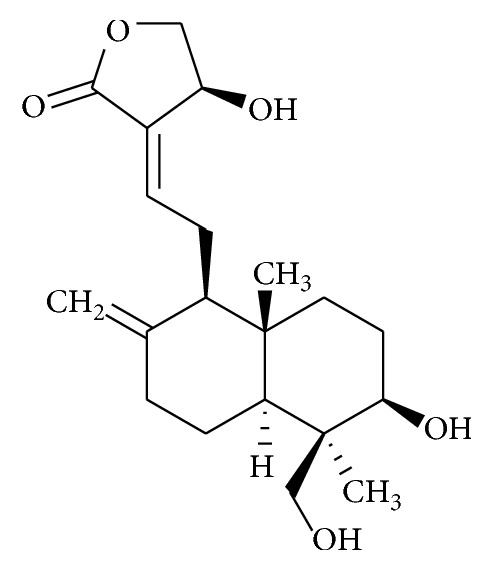
Chemical structure of andrographolide.

The andrographolide was purchased from Sigma-Aldrich (St. Louis, Missouri, USA), the purity was over 98%, and this was sponsored by Korea Bioactive Natural Material Bank (KBNMB). The molecular structure of andrographolide is shown in Figure 1. Cell culture media, FBS, and P/S were purchased from Hyclone (GE Healthcare Life Sciences, Little Chalfont, UK). The antibodies were purchased from Abcam (Cambridge, MA, USA), Cayman (Ann Arbor, MI, USA), or Santa Cruz Biotechnology (Dallas, TX, USA). Most of chemicals and reagents used in the present study were American Chemical Society grade.

### 2.3. 3D Molecular Docking Simulation

3D molecular docking simulation of andrographolide into bric-a-brac/tram-track/broad (BTB) complex domain of Keap1 was performed based on the CHARMm force fields algorithm* in silico* by using Discovery Studio 4.0 (Accelys, San Diego, CA, USA). The active site 1 of BTB domain of Keap1 (PDB code: 4CXT, 4CXT with mutant at C151W) was subjected to calculate the CDOCKER interaction energy. The structures of andrographolide and Keap1 were used after rearrangement by “clean protein” or “clean geometry.” The pH of protein was adjusted at 7.4. The negative value of CDOCKER interaction energy indicates high binding capacity.

### 2.4. Real-Time PCR

The mRNA was extracted from HT22 cells using the Trizol method, and extracted mRNA was immediately synthesized to cDNA using Maxim RT PreMix (random primer) manufactured from iNtRON Biotechnology (Seongnam, Korea). Approximately 500 *μ*g of synthesized cDNA was used in real-time PCR. HO-1 and 18S primers were designed as indicated below. Primers used in this study were HO-1 (forward; CAGCCCCACCAAGTT, reverse; GGCGGTCTTAGCCTCTTCTGT) and 18S (forward; GCTTAATTTGACTCAACACGGGA, reverse; AGCTATCAATCTGTCAATCCTGTC). Real-time PCR was performed using the StepOne Real-Time PCR System (Thermo Fisher Scientific, Waltham, MA, USA) with 2X SYBR green and ROX dye (Bioneer, Daejeon, Korea). The mixtures were placed in a StepOne Real-Time PCR System for 10 min incubation at 95°C and 40 cycles of 15 s at 95°C and 1 min at 60°C. The relative mRNA expression levels were calculated using the threshold cycle number (ΔΔC_t_ value).

### 2.5. Western Blot Analysis

The cells were treated with various concentrations of andrographolide and then cells were incubated. The treatment concentrations of samples were indicated in each figure legend. After the incubation, the cells were rinsed twice with cold phosphate buffered saline (PBS). Subsequently, the nuclear extracts were isolated from cells using a subcellular protein fractionation kit (Thermo scientific, Rockford, IL, USA). Whole cell lysates were extracted using EBC lysis buffer [120 mM NaCl, 0.5% Nonidet P-40, 5 *μ*g/mL leupeptin, 10 *μ*g/mL aprotinin, 50 *μ*g/mL phenylmethane sulfonyl fluoride, 0.2 mM sodium orthovanadate, 100 mM NaF, and 50 mM Tris-Cl (pH 8.0)] and isolated by centrifugation at 12,000 ×g for 10 min at 4°C. The protein contents were calculated using the bicinchoninic acid (BCA) protein quantification method (Bio-rad, Hercules, CA, USA). Sodium dodecyl sulfate-polyacrylamide gel electrophoresis was performed using an electrophoresis kit obtained from Bio-rad (Hercules, CA, USA). A total of 30 *μ*g of proteins were loaded onto the gel and run at 50 V for 30 min, and the voltage of the power supply was changed to 200 V for 1 h. The proteins were transferred to Immobilon®-P 0.45 *μ*m polyvinylidene difluoride transfer membrane from Millipore (Billerica, MA, USA). The nonspecific proteins were soaked in 5% skim milk dissolved in tris-buffered saline (TBS)/0.2% Tween20® (T) for 1 h at room temperature for blocking, and subsequently, the membranes were rinsed three times with TBS/T. The membranes were incubated with primary antibodies, including anti-Nrf2 (1 : 1000), anti-HO-1 (1 : 1000), anti-pNF-*κ*B (1 : 1000), anti-COX-II (1 : 1000), anti-i-NOS (1 : 1000), anti-Lamin B (1 : 2000), or anti-*β*-actin (1 : 2000), for 3.5 h at room temperature or overnight at 4°C, with slight digitation. After washing the membranes three times with TBS/T for 10 min each time, the membranes were treated with secondary antibodies, including horseradish peroxidase (HRP)-conjugated anti-rabbit, anti-goat, or anti-mouse-immunoglobulin G, for 2 h at room temperature. After washing the membranes three times with TBS/T for 10 min each time, the membranes were treated with chemiluminescent substrates (Thermo Scientific, Rockford, IL, USA). The target proteins were visualized in using the ImageQuant^TM^ LAS4000 imaging system from GE Healthcare Life Sciences (Little Chalfont, UK). The relative protein expression levels were calculated using ImageJ software (National Institutes of Health, Bethesda, MD, USA).

### 2.6. ARE-Reporter Gene Assay

HT22 cells were transfected with the pGL4.37[luc2P/ARE/Hyg] vector (Promega Corp., Madison, WI, USA) and manufactured by Jong-Sang Kim's laboratory from Kyungpook National University to generate the stable cell line “HT22-ARE” [17]. Briefly, the cells were seeded at 3 × 10^5^ cells per well onto 12-well plates and starved in DMEM/high-glucose supplemented with 1% FBS and P/S for 12 h. Subsequently, the cells were treated with each sample for 16 h. The cells were lysed in 120 *μ*L of passive lysis buffer for thirty minutes with digitation every 10 min. The cell lysates were isolated by the centrifugation at 12,000 ×g for 2 min. The supernatant was used to measure luminescence using a SpectraMax L microplate reader (Molecular Devices, San Francisco, CA, USA). The ARE-luciferase activity was expressed to the relative induction compared with the control.

### 2.7. Preparation of A*β*_42_ Construct

The plasmids for overexpressing A*β*_42_, containing an enhanced green fluorescent protein (pEGFP-C1) and kanamycin resistant genes, were gifted from Professor Junsoo Park at Yonsei University. The cells were transfected with A*β*_42_ plasmids using Lipofectamine 2000 (Invitrogen, Carlsbad CA, USA) for 12 h according to the manufacturer's instructions.

### 2.8. Immunocytochemistry

HT22 cells were plated onto 35 mm cell culture plates at a density of 3 × 10^5^ cells/plate. After confirmation of cell attachment, the cells were treated with various concentrations of andrographolide dissolved in low-serum medium containing 1% FBS and P/S for 12 h, followed by fixation with 3.7% formalin solution for a week. The cell plasma membranes were permeabilized after soaking in 0.2% Triton X-100 solution in PBS, and the permeabilized cells were blocked with 1% bovine serum albumin solution. The cells were incubated with primary antibodies (1 : 200 ratio) overnight at 4°C. After discarding the solution with primary antibodies and washing with PBS five times, the cells were incubated with secondary antibodies, including anti-rabbit fluorescein isothiocyanate (1 : 500 ratio) and anti-goat Texas red (1 : 500 ratio), for 2 h at room temperature. Subsequently, the cells were stained with 1 *μ*g/mL 4′,6-diamidino-2-phenylindole (DAPI) solution for 1 min. The cells were washed five times with PBS and mounted using Vectashield mounting medium (Vector Laboratories, Burlingame, CA, USA). The immunofluorescence images were captured using confocal microscopy TCS SP8 (Leica, Wetzlar, Germany).

### 2.9. Cytokine Assay

The levels of IL-6, IL-1*β*, or PGE_2_ secreted from BV-2 cells were analyzed using mouse IL-6 (BD Biosciences, San Jose, CA, USA), mouse IL-1*β* (Thermo Scientific, Rockford, IL, USA), and PGE_2_ (Thermo Scientific, Rockford, IL, USA) ELISA kits, respectively, according to the manufacturer's instructions, with slight modifications. Briefly, the conditioned medium collected from BV-2 cells plated in 60 mm cell culture dishes was transferred to coated strip-type 96-well plates and incubated for 2 h at room temperature. The medium was removed and biotinylated anti-IL-6 or anti-IL-1*β* antibodies were added and incubated for 1 h. The plates were washed with wash buffer, and streptavidin HRP solution was then added to each well, followed by incubation for 30 min at room temperature. After washing the plates with wash buffer, 3,3′,5,5′-tetramethylbenzidine substrates were added, followed by incubation for another 30 min and the subsequent addition of stop solution to each well. The absorbance was measured at 450 nm. For PGE_2_ determination, the medium was added to each well of the immune-plate provided in the kit. The PGE_2_-conjugate and PGE_2_ antibody were added to the wells and incubated for 2 h with strong agitation at 300 rpm. The wells were washed three times with wash buffer, and substrate solution was added, followed by incubation for 45 min and subsequent detection at 405 nm using the VersaMax^TM^ microplate reader (Biocompare, San Francisco, CA, USA). The secreted cytokine levels were expressed as a percentage of the control value.

### 2.10. NO Assay

BV-2 cells were plated onto 60 mm plates at 95% confluency and treated with A*β*_42_ transfectant for 12 h. The medium was replaced with phenol red and serum-free medium containing various concentrations of andrographolide. After 24 h, the medium was used to measure NO production at 540 nm using the VersaMax^TM^ microplate reader (Biocompare, San Francisco, CA, USA).

### 2.11. Statistical Analysis

Statistical calculations were conducted using analysis of variance (ANOVA), followed by Tukey's range test in the SPSS Statistics 23 software (SPSS, Inc., Chicago, IL, USA). The data are presented as the means ± standard error (SE) of triplicates. The significance presented as ^*∗*^*p* < 0.05, ^*∗∗*^*p* < 0.01, and ^*∗∗∗*^*p* < 0.001 compared to control group, ^+++^*p* < 0.001 for comparison with the brusatol-untreated group, ^#^*p* < 0.05, ^##^*p* < 0.01, and ^###^*p* < 0.001 compared to the A*β*_42_-transfected group.

## 3. Results and Discussion

### 3.1. Andrographolide Upregulates Nrf2 Expression and Nuclear Translocation

**Figure 2:**
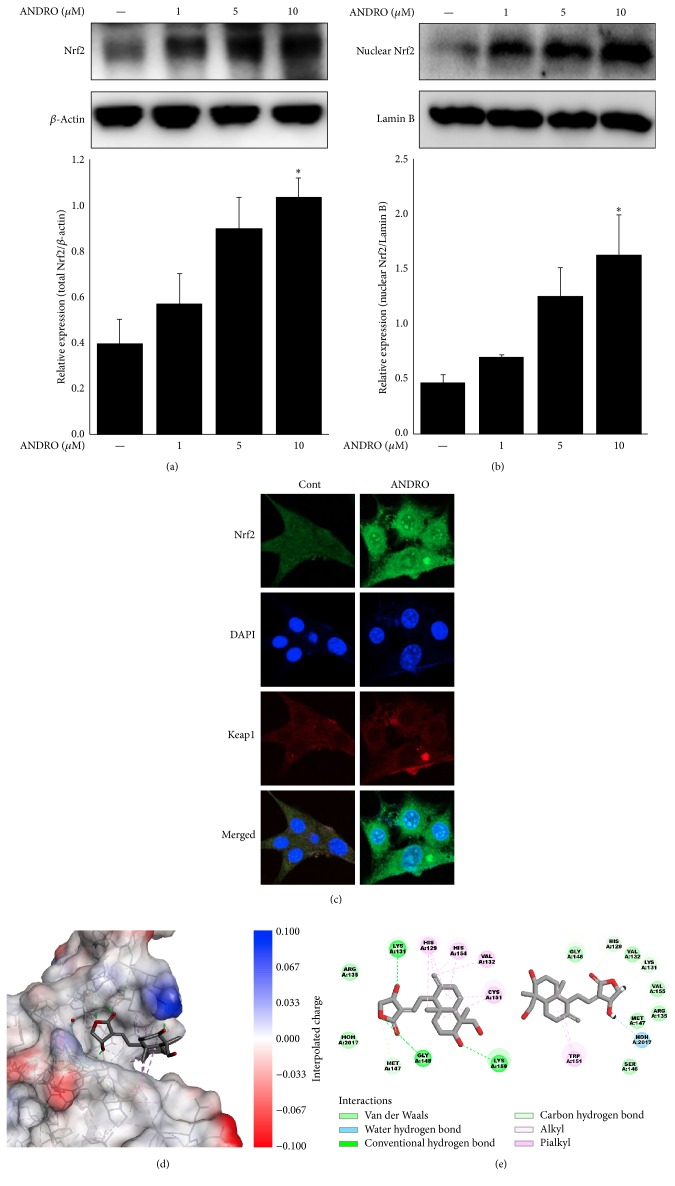
Effect of andrographolide on the expressions and locations of Nrf2 and Keap1. The abbreviation of andrographolide, ANDRO, was used. (a) Total Nrf2 expression was determined by Western blot analysis. The cells were treated with 1, 5, and 10 *μ*M andrographolide for 24 h and the cell lysates were subjected to Western blot analysis. The data are presented as the means ± SE of triplicates. The significance presented as ^*∗*^*p* < 0.05 compared with control group. (b) Nuclear Nrf2 expression was determined by Western blot analysis. Lamin B was used as a nuclear loading control. The data are presented as the means ± SE of triplicates. The significance presented as ^*∗*^*p* < 0.05 compared with control group. (c) The intracellular location and deposition of Nrf2 and Keap1 were examined using immunocytochemistry. The cells were plated and incubated with 10 *μ*M andrographolide for 24 h. Cells were immediately fixed with 3.7% formalin solution and subjected to the next steps described in the “Materials and Methods” section. (d) 3D molecular docking simulation of andrographolide to BTB domain of Keap1 was monitored* in silico*. The Keap1 protein was visualized by interpolated charge. (e) 2D diagram for non-covalent interactions between andrographolide and wild type or C151W mutated Keap1 was presented.

To investigate the role of Nrf2 in response to andrographolide treatment in the murine hippocampal HT22 cell line, the expression pattern of nuclear Nrf2 was evaluated by Western blot analysis. In the case of the Nrf2 protein expression levels, andrographolide treatment at 10 *μ*M significantly increased total Nrf2 and nuclear Nrf2 expression levels up to 2.6 and 3.4 times than normal cells, respectively (Figures 2(a) and 2(b)). Moreover, andrographolide upregulated the expression of Nrf2 in both the cytoplasm and nucleus and induced the translocation of Nrf2 from the cytoplasm to the nucleus. The results of immunocytochemistry experiments showed no changes in the protein expression of Keap1, an inhibitory protein of Nrf2 [17] after treatment with 10 *μ*M of andrographolide (Figure 2(c)).

### 3.2. Andrographolide Interacts Strongly with C151 in BTB Domain of Keap1

Keap1, well-known inhibitor protein of Nrf2, is composed of heterodimeric complex binding to Nrf2. Ubiquitination of Nrf2-Keap1 complex under normal condition promotes proteasome degradation. Recently, to the efforts of searching for cytoprotective agents, the study about Keap1 as therapeutic target has been reported. The BTB domain of Keap1 acts as the sensor to electrophiles; thereby these disrupt Nrf2-Keap1 complex through conformational change of Keap1. Specifically, the covalent modification in cysteine residues in BTB domain can change the protein conformation; among the cysteine residues including C151, C273, and C288, C151 is one of the candidates to disrupt Nrf2/Kelch interaction of Keap1 conformation [18].  Figure 2(d) shows the docking pose of andrographolide into the BTB domain of Keap1. The results showed that non-covalent bonds including alkyl, pialkyl, and hydrogen bond were formed between andrographolide and BTB domain. Andrographolide interacted with H129, V132, C151, and H154 by pialkyl or alkyl bond and also interacted with K131, G148, and K150 by hydrogen bond. To investigate the involvement of C151 in andrographolide-Keap1 interaction, C151 was mutated to W151* in silico*. The 3D configuration of Keap1 was slightly changed by C151W mutation (Figure 2(e)). As a result, CDOCKER interaction energies of wild type and C151W mutated Keap1 with andrographolide revealed −31.6 ± 1.2 and −21.5 ± 4.4 kcal/mole, respectively. The differences between values of CDOCKER interaction energies from the results were significant (*p* < 0.001). In addition, as presented in Figure 2(e), most of non-covalent bonds such as pialkyl, alkyl, and hydrogen bonds were diminished due to C151W mutation. These results suggest that C151 in BTB domain of Keap1 has critical role in the interaction of andrographolide with Keap1.

### 3.3. Andrographolide Activates the Transcription of ARE Gene Modulated by Nrf2

**Figure 3:**
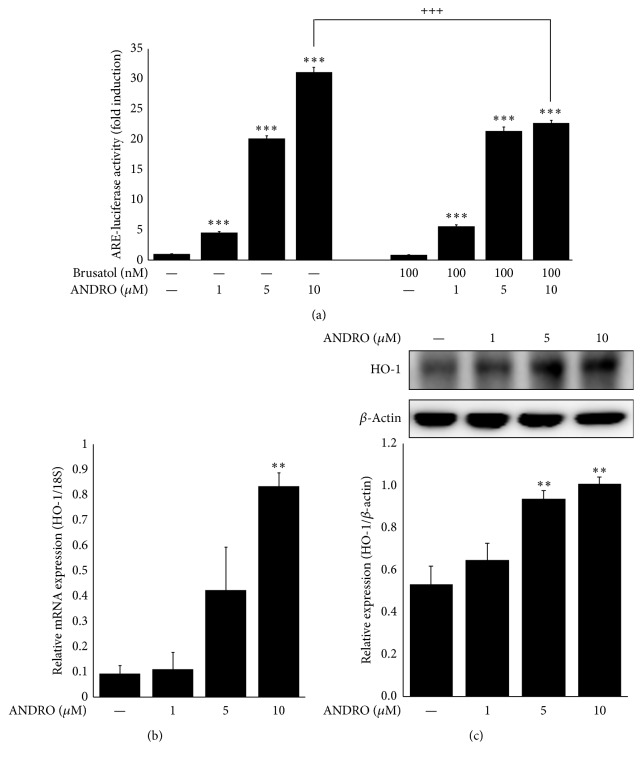
Effect of andrographolide on ARE gene transcriptional activity and HO-1 expressions. The abbreviation of andrographolide, ANDRO, was used. (a) HT22-ARE cells were treated with 100 nM brusatol for 2 h prior to andrographolide treatment at 1, 5, or 10 *μ*M concentrations for 16 h. The cells were lysed, and the lysates were used to determine luciferase activity. The data are presented as the means ± SE of triplicates. The significance presented as ^*∗∗∗*^*p* < 0.001 compared with control group, ^+++^*p* < 0.001 compared with the brusatol-untreated group. (b) Relative mRNA expression levels of Nrf2 were regulated after andrographolide treatment. HT22 cells were seeded onto 12-well plates at 1.5 × 10^5^ cells per a well and treated with 1, 5, and 10 *μ*M andrographolide for 24 h. The mRNA was extracted, cDNA was synthesized, and the relative expression levels of mRNA were determined. (c) HO-1 expression after andrographolide treatment at 1, 5, or 10 *μ*M concentrations for 24 h in HT22 cells was determined by Western blot analysis. The data are presented as the means ± SE of triplicates. The significance presented as ^*∗∗*^*p* < 0.01 compared with control group.

As andrographolide docked into the Keap1 and also induced the Nrf2 translocation into nucleus, we are trying to test ARE transcriptional activity by andrographolide. As shown in Figure 3(a), the treatment of andrographolide increased ARE-mediated transcription at 1, 5, and 10 *μ*M in a concentration-dependent manner. However, these activities were sharply diminished to approximately two-thirds the initial levels at andrographolide treated-cells after pretreatment with 100 nM of brusatol which was used a Nrf2 inhibitor. These data indicate that andrographolide activates ARE-mediated transcription through Nrf2 activation. According to the literature, PKC activates Nrf2 by phosphorylation at Ser-40, and phosphorylated Nrf2 subsequently regulates ARE-mediated transcription [19]. The activation of cytoprotective enzymes in response to reactive electrophiles or phytochemicals is initially regulated at the transcriptional level following the central dogma theory. Primarily this transcriptional activation is mediated by a* cis*-element of ARE gene, and thereby the regulation of cellular redox homeostasis involved in this ARE gene is critical point to understand the action mechanism of phytochemicals [20].

### 3.4. Andrographolide Increases the Expression of HO-1 in HT22 Cells

HO-1 is one of representative cytoprotective enzymes; therefore, to investigate the effect of andrographolide on the mRNA and protein expression of HO-1, a downstream antioxidant enzyme of Nrf2, the cells were incubated with andrographolide at a concentration range of 1 to 10 *μ*M for 24 h. The mRNA expression was upregulated 9.0 times after andrographolide treatment at 10 *μ*M compared with the untreated control, and the protein expression levels of HO-1 were significantly increased up to 1.9 times after andrographolide treatment at 10 *μ*M (Figures 3(b) and 3(c)). HO-1 is the inducible and rate-limiting enzyme of heme degradation. Moreover, the induction of HO-1 protects against the cytotoxicity resulting from oxidative stress and exerts inflammation inhibitory properties [21]. Thus, HO-1 is considered a marker enzyme for monitoring the therapeutic response to neuroprotection or anti-inflammation. Among the inflammations, neuroinflammation is mainly observed in the damaged brain and microglia are in charge of the neuroinflammatory process as part of the operation for the innate immune system. For the next step, the involvement of Nrf2, which is a transcription factor of HO-1, in microglial activation and A*β* clearance was studied.

### 3.5. Andrographolide Likely Diminished Microglial A*β*_42_ Prevalence Partially through Nrf2

**Figure 4:**
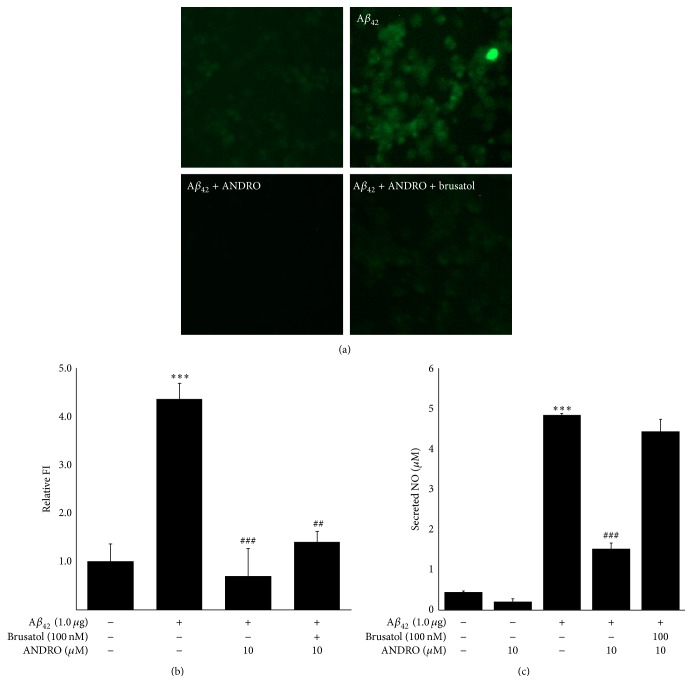
Effect of andrographolide and/or brusatol on A*β*_42_ expression in BV-2 cells. BV-2 cells were transfected with A*β*_42_ plasmids for 12 h. The medium was discarded and replaced with phenol red-free medium in the absence or presence of 100 nM brusatol. After 2 h, the medium was replaced with fresh medium containing 1, 5, or 10 *μ*M of andrographolide. The abbreviation of andrographolide, ANDRO, was used. (a) The microglial clearance of A*β*_42_ was visualized by fluorescence microscope imaging. (b) The relative fluorescence intensity of A*β*_42_ was quantified by Image J software. The data are presented as the means ± SE of triplicates. The significance presented as ^*∗∗∗*^*p* < 0.001 compared with control group and ^##^*p* < 0.01 and ^###^*p* < 0.001 compared with the A*β*_42_-transfected group. (c) The secreted NO levels in the medium of BV-2 cells were measured using the Griess method. The data are presented as the means ± SE of triplicates. The significance presented as ^*∗∗∗*^*p* < 0.001 compared with control group and ^###^*p* < 0.001 compared with the A*β*_42_-transfected group.

Microglia are typically present in the white and gray matter of the brain and migrate throughout the neuronal tissues. These cells play dual roles in A*β* pathogenesis, including elimination of A*β* aggregation through phagocytosis and the release of neurotoxic proteases and inflammatory factors by facilitating A*β* accumulation [22]. Recently, the importance of apolipoprotein E in A*β* pathogenesis was demonstrated, which, in turn, promotes A*β* trafficking and intracellular A*β* degradation [23]. As shown in Figure 4, enhanced green fluorescence protein- (EGFP-) tagged A*β*_42_ peptide was observed in BV-2 cells after the transient transfection of A*β*_42_ plasmid to cells. Green fluorescence was reduced after andrographolide treatment at 10 *μ*M. In the case of A*β*_42_-overexpressed cells, pretreatment with brusatol prior to andrographolide treatment increased slightly the intensity of green fluorescence compared to the andrographolide treatment group. These results suggest that andrographolide clears intracellular A*β*_42_ peptides; however, this might only be a partial effect through Nrf2 activation based on the results shown in Figure 4. The possible postulation to explain this phenomenon is A*β* clearance involved in A*β*-degrading enzymes. For example, glutamate carboxypeptidase II, matrix metalloproteinase 9, and insulin-degrading enzyme are closely associated with A*β* peptide clearance [24]. Consistently, the elevated production of NO by transient A*β*_42_ transfection was reduced after andrographolide treatment, and this phenomenon was reversed after pretreatment with brusatol. Considering that NO induction is observed in microglial activation [25], these data suggest that the inhibition of A*β*_42_-induced NO production by andrographolide treatment could be associated with Nrf2 mediation. Furthermore, this A*β* peptide clearing mechanism is considered a potent therapeutic tool for targeting AD [26] and suggests the therapeutic potential of andrographolide as an anti-AD drug that removes A*β* peptides.

### 3.6. Andrographolide Downregulates pNF-*κ*B-Mediated COX-II and i-NOS Expression Induced by A*β*_42_ Plasmid Transfection in Microglial BV-2 Cells

**Figure 5:**
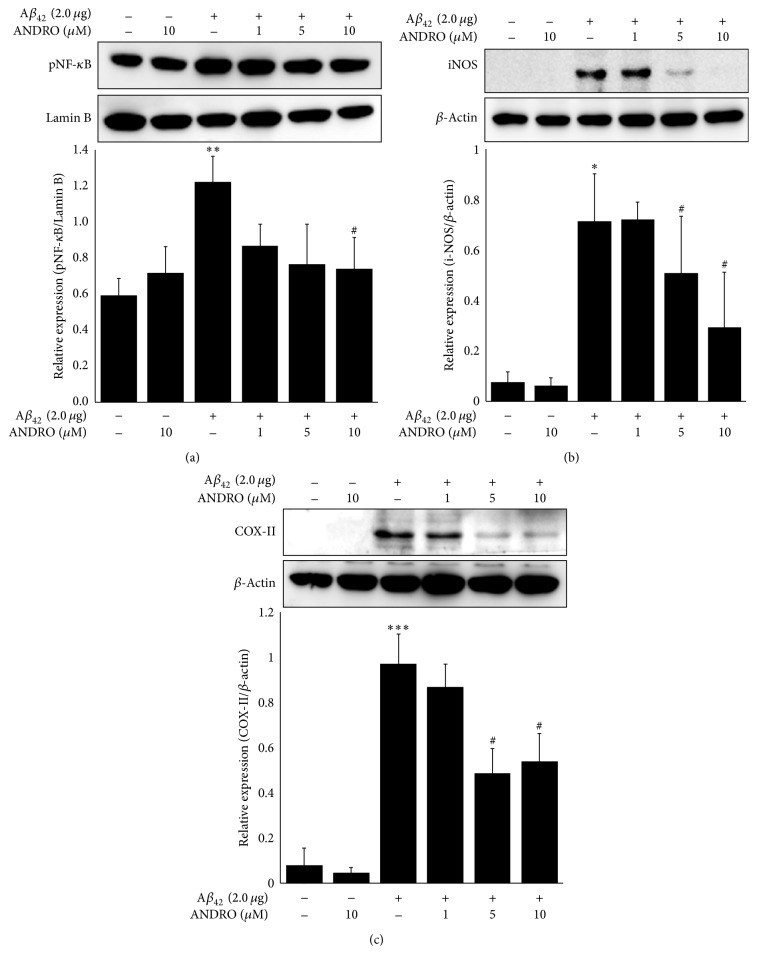
Effect of andrographolide on pNF-*κ*B or inflammatory protein expression in BV-2 cells. BV-2 cells were seeded and transfected with A*β*_42_ plasmids for 12 h, and the medium was then replaced with fresh phenol red free medium containing 1, 5, or 10 *μ*M of andrographolide for 24 h. The abbreviation of andrographolide, ANDRO, was used. (a) Nuclear extracts were used to determine pNF-*κ*B (Ser536) accumulation in the nucleus. Lamin B was used as a positive control. The data are presented as the means ± SE of triplicates. The significance presented as ^*∗∗*^*p* < 0.01 compared with control group and ^#^*p* < 0.05 compared with the A*β*_42_-transfected group. (b) Whole cell lysates were used to analyze i-NOS expression, determined by Western blot analysis. The data are presented as the means ± SE of triplicates. The significance presented as ^*∗*^*p* < 0.05 compared with control group and ^#^*p* < 0.05 compared with the A*β*_42_-transfected group. (c) COX-II expression was analyzed by Western blot analysis. The data are presented as the means ± SE of triplicates. The significance presented as ^*∗∗∗*^*p* < 0.001 compared with control group and ^#^*p* < 0.05 compared with the A*β*_42_-transfected group.

To evaluate the effect of andrographolide on pNF-*κ*B accumulation in the nucleus, microglial BV-2 cells were transfected with 2.0 *μ*g of A*β*_42_ and treated with 1 to 10 *μ*M of andrographolide. A*β*_42_ activated the pNF-*κ*B signaling pathway and induced inflammatory responses in microglia cells. As shown in Figure 5(a), pNF-*κ*B (Ser536) accumulated in the nucleus when BV-2 cells were transfected with 2.0 *μ*g of A*β*_42_, and the nuclear accumulation of pNF-*κ*B was dramatically decreased after andrographolide treatment by concentrations. Mainly, pNF-*κ*B RelA/p65-p50 heterodimer is transported to nucleus after releasing from I*κ*B*α* and phosphorylation at Ser536 [27]. Our results showed that pNF-*κ*B accumulation in nucleus was suppressed by andrographolide treatment; however their specific mechanism is not yet clarified. Therefore, further investigation for underlying mechanism to regulate pNF-*κ*B machinery by andrographolide treatment is demanded. On the other hands, in the case of downstream inflammatory proteins regulated by pNF-*κ*B including i-NOS and COX-II, the cellular expression was increased after A*β*_42_ transfection, whereas andrographolide treatment at 10 *μ*M significantly decreased the expression of i-NOS and COX-II (Figures 5(b) and 5(c)).

### 3.7. Andrographolide Inhibits the Secretion of IL-1*β*, IL-6, PGE_2_, and NO in BV-2 Cells

**Figure 6:**
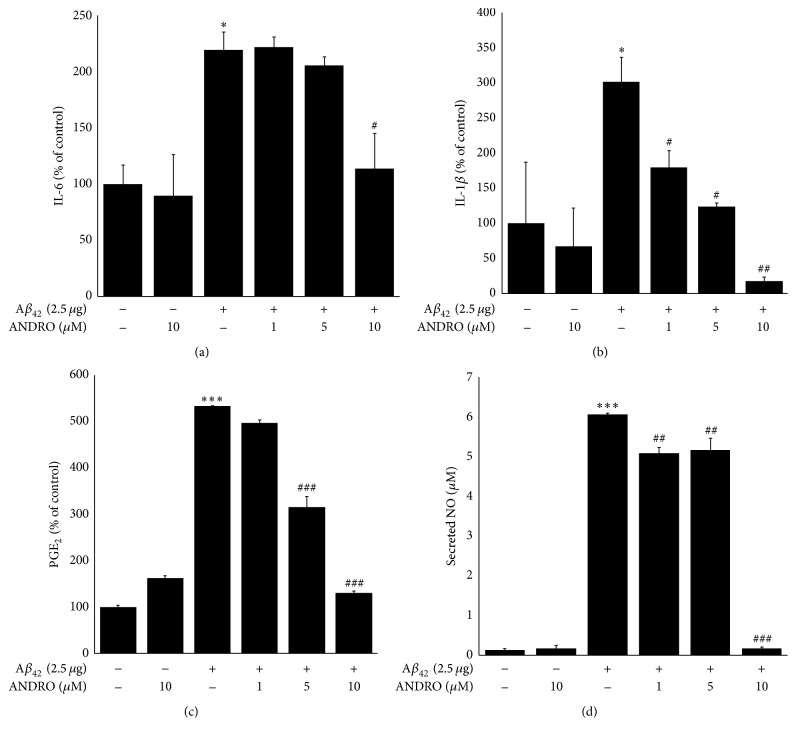
Effect of andrographolide on the secretion inflammatory cytokines, PGE_2_ and NO. BV-2 cells were seeded onto culture dishes and transfected with A*β*_42_ plasmids for 12 h. Subsequently, the medium was replaced with fresh phenol red-free medium containing various concentrations of andrographolide for 24 h. The conditioned medium was collected and used to analyze the secretion of IL-1*β*, IL-6, PGE_2_, and NO. The abbreviation of andrographolide, ANDRO, was used. The data are presented as the means ± SE of triplicates. The significance presented as ^*∗*^*p* < 0.05 and ^*∗∗∗*^*p* < 0.001 compared with control group and ^#^*p* < 0.05, ^##^*p* < 0.01, and ^###^*p* < 0.001 compared with the A*β*_42_-transfected group.

Because inflammatory cytokines are commonly regulated by NF-*κ*B-mediated signaling pathway, we further evaluated the cytokines, PGE_2_, and NO levels. To determine the effect of andrographolide on inflammatory cytokines and nitric oxide levels in microglial BV-2 cells, the conditioned medium was collected and subjected to perform cytokine and PGE_2_ and NO assays. As presented in Figures 6(a) and 6(b), A*β*_42_ significantly and dramatically increased the levels of IL-1*β* or IL-6 in BV-2-conditioned medium. These IL-1*β* or IL-6 levels were significantly reduced after the treatment of BV-2 cells with andrographolide at 10 *μ*M. In particular, IL-1 is considered a master regulator of neuroinflammation [28]. IL-1*β*, referred to as a pyrogenic cytokine [29], increases IL-1*β* production in the brain of AD patients [30]. The increased IL-1*β* regulates intracellular A*β* synthesis, thereby contributing to cellular damage [31]. Therefore, it is meaningful to reduce the levels of secreted IL-1*β* levels from BV-2 cells after andrographolide treatment. In the case of PGE_2_ and NO levels, the treatment of andrographolide decreased the induction of secreted PGE_2_ levels after A*β*_42_ transfection in the conditioned medium of BV-2 cells in a concentration-dependent manner. To assess the final step of neuroinflammation, secreted NO levels were measured using a spectrophotometric method. The increased NO content in BV-2-conditioned medium after A*β*_42_ plasmid transfection was reversed after andrographolide treatment. Because microglial-mediated neuroinflammation is attributed to AD progression [2], the anti-neuroinflammatory effects of andrographolide suggest its therapeutic potential for the development of a drug targeting for AD.

## 4. Conclusion

In summary, andrographolide activated the Nrf2/Keap1-mediated HO-1 signaling pathway in murine hippocampal HT22 cells, promoted the clearance of intracellular A*β*_42_ peptides in BV-2 cells, and downregulated the pNF-*κ*B signaling pathway. Moreover, andrographolide exerts neuroinflammation inhibitory activity. Thus, further investigation of the roles of andrographolide in AD would be worthy.

## Supplementary Material

Repetition of biological data.
