# Supplementary material for: Andrographolide Activates Keap1/Nrf2/ARE/HO-1 Pathway in HT22 Cells and Suppresses Microglial Activation by Aβ42 through Nrf2-Related Inflammatory Response
Source: Mediators Inflamm. 2017 Mar 8;2017:5906189. doi: 10.1155/2017/5906189 (PMC5360972; doi:10.1155/2017/5906189)
Supplement: Supplementary file 1 — Repetition of biological data. [file 5906189.f1.doc]

**Appendix A. Supplementary data**

**Andrographolide activates Keap1/Nrf2/ARE/HO-1 pathway in HT22 cells and suppresses microglial activation by Aβ_42_ through Nrf2-related inflammatory response**

Ji Yeon Seo^1^, Euisun Pyo^1^, Jin Pyo An^1^, Jinwoong Kim^1^, Sang Hyun Sung^1^, Won Keun Oh^1,*^

^1^ Korea Bioactive Natural Material Bank, Research Institute of Pharmaceutical Sciences, College of Pharmacy, Seoul National University, Seoul 08826, Republic of Korea

*Correspondence : wkoh1@snu.ac.kr

Seoul National University, Seoul 08826, Republic of Korea

Tel and Fax: +82-02-880-7872. E-mail: wkoh1@snu.ac.kr.

**Figure Captions**

**Fig. S1.** Nuclear Nrf2 expression by andrographolide in HT22 cells.

**Fig. S2.** The regulation of Nrf2 or HO-1 expressions by andrographolide in HT22 cells.

**Fig. S3.** The intracellular Nrf2 or Keap1 in HT22 cells.

**Fig. S4.** The nuclear pNFκB expression in BV-2 cells.

**Fig. S5.** The regulation of iNOS or COX-II expressions by andrographolide in BV-2 cells.

**Fig. S6.** The change of cell morphology and Aβ_42_ expression by andrographolide in the absence or presence of brusatol in BV-2 cells.

**Fig. S1.** Nuclear Nrf2 expression by andrographolide in HT22 cells.

Nuclear extract of cells were used to determine nuclear Nrf2 expression level. The experiments were performed by Western blot analysis. N=3.

**
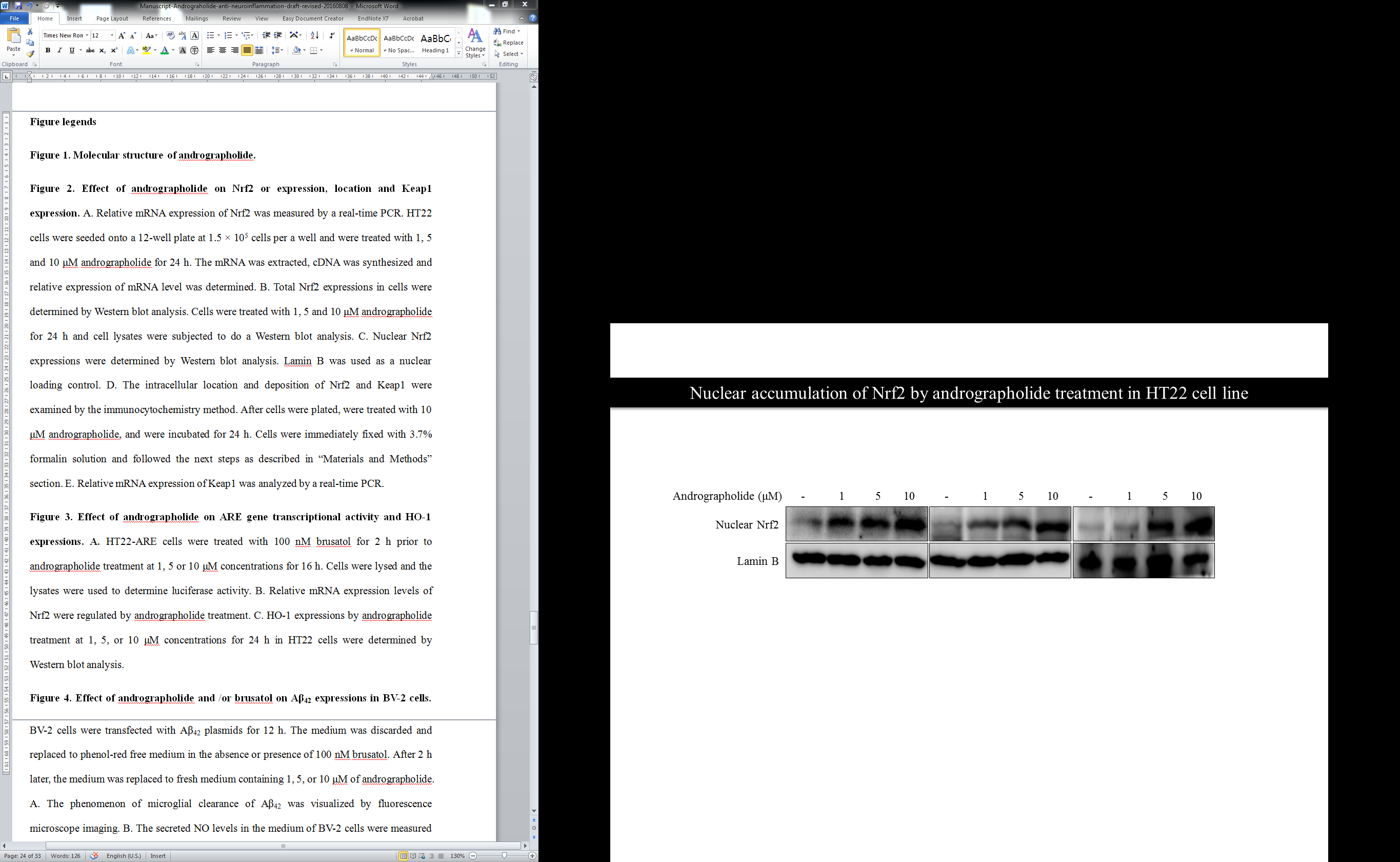
**

**Fig. S2.** The regulation of Nrf2 or HO-1 expressions by andrographolide in HT22 cells. Whole cell lysate was collected and prepared to do the Western blot analysis. N=3.

**
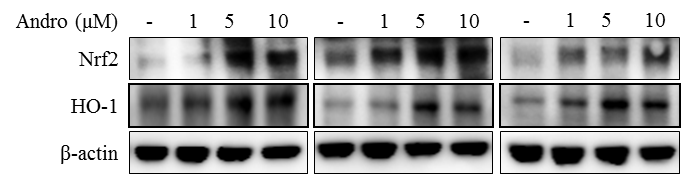
**

**Fig. S3.** The intracellular Nrf2 or Keap1 in HT22 cells.

Cells were immuno-reacted with fluorescence-tagged antibodies. The image was visualized by Confocal microscope.

**
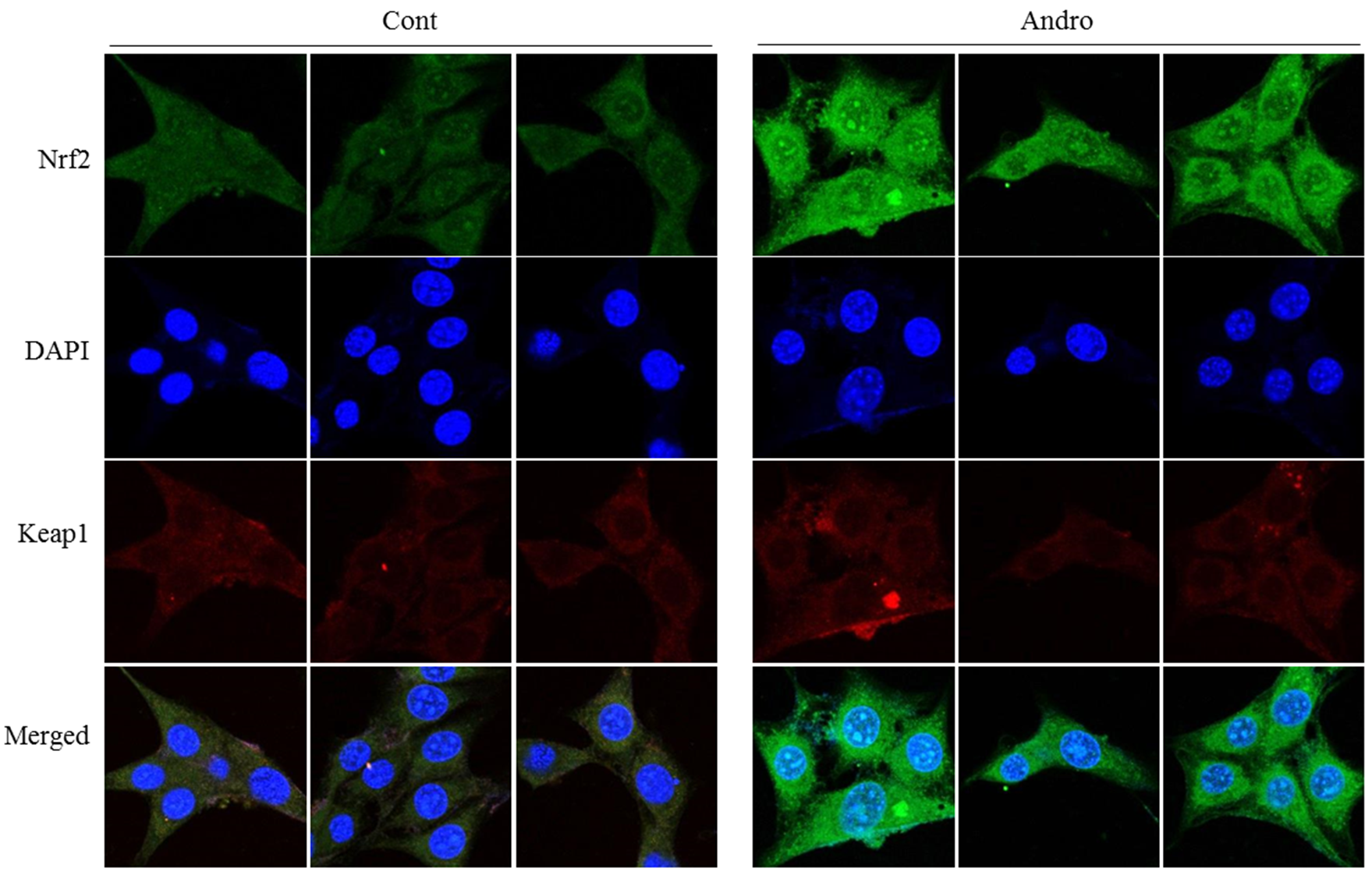
**

**Fig. S4.** The nuclear pNFκB expression in BV-2 cells.

The nuclear extract was collected and prepared to do Western blot analysis.

**
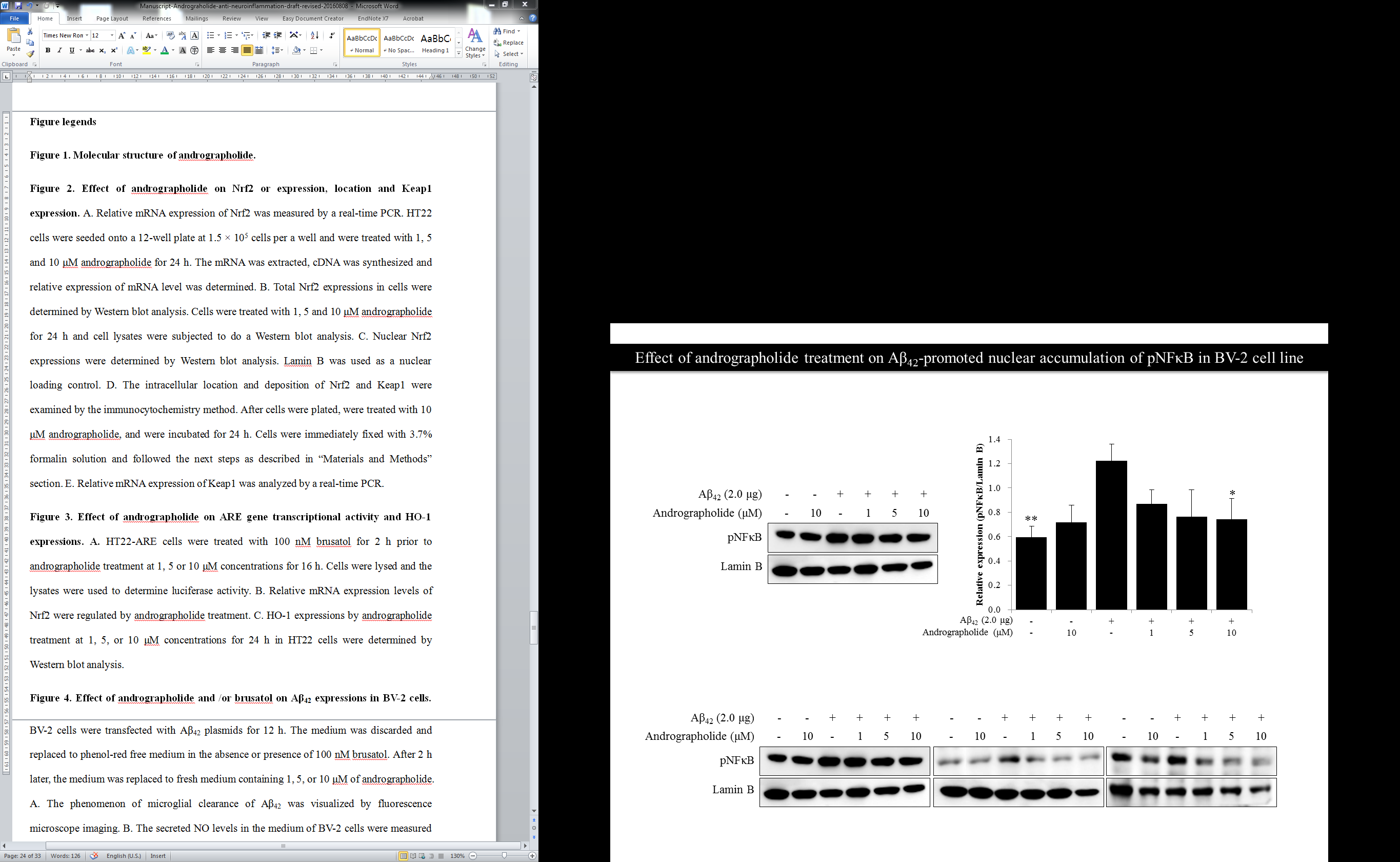
**

**Fig. S5.** The regulation of iNOS or COX-II expressions by andrographolide in BV-2 cells. Whole cell lysate was collected and prepared to do the Western blot analysis. N=3.

**
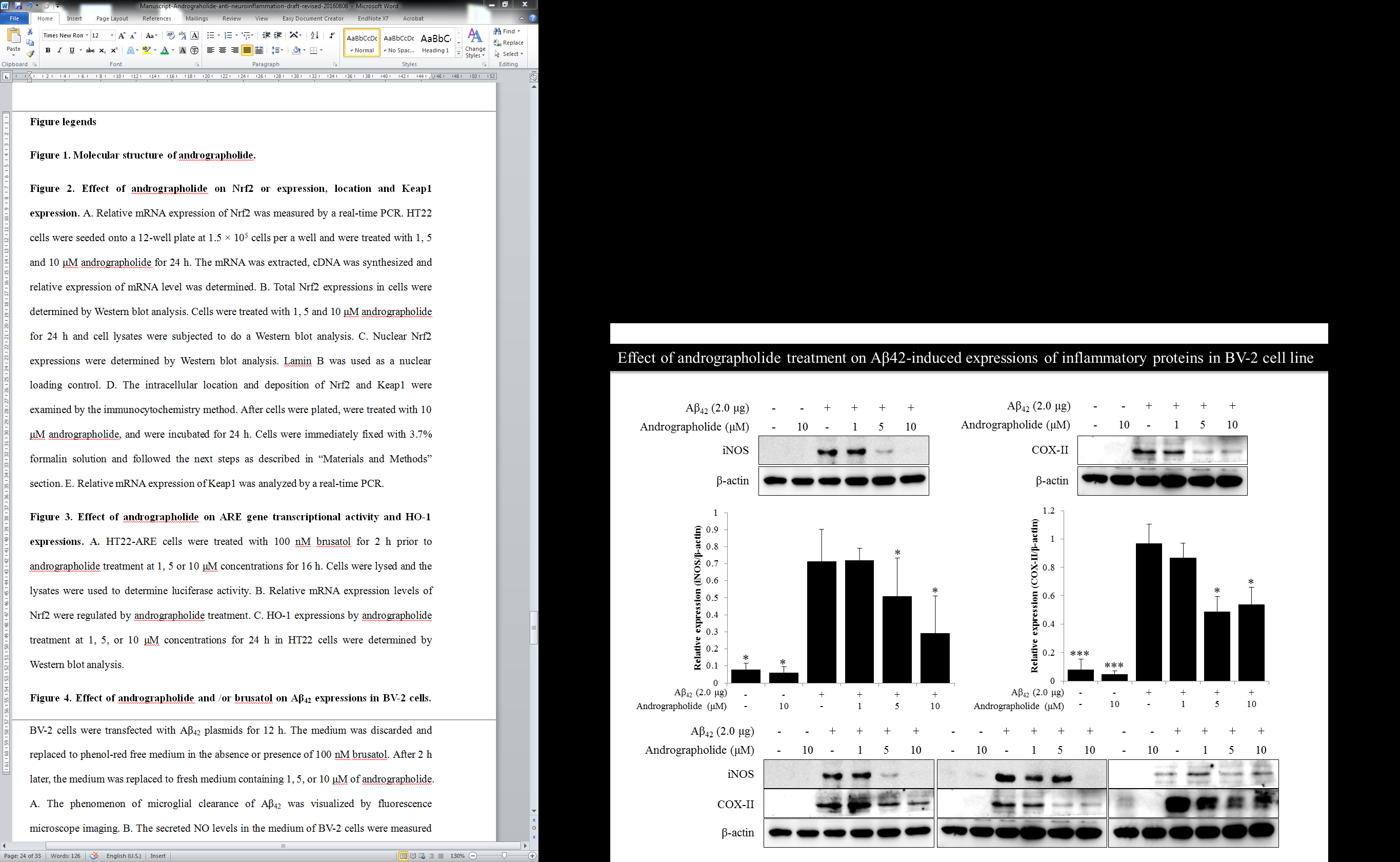
**

**Fig. S6.** The change of cell morphology and Aβ_42_ expression by andrographolide in the absence or presence of brusatol in BV-2 cells.

**
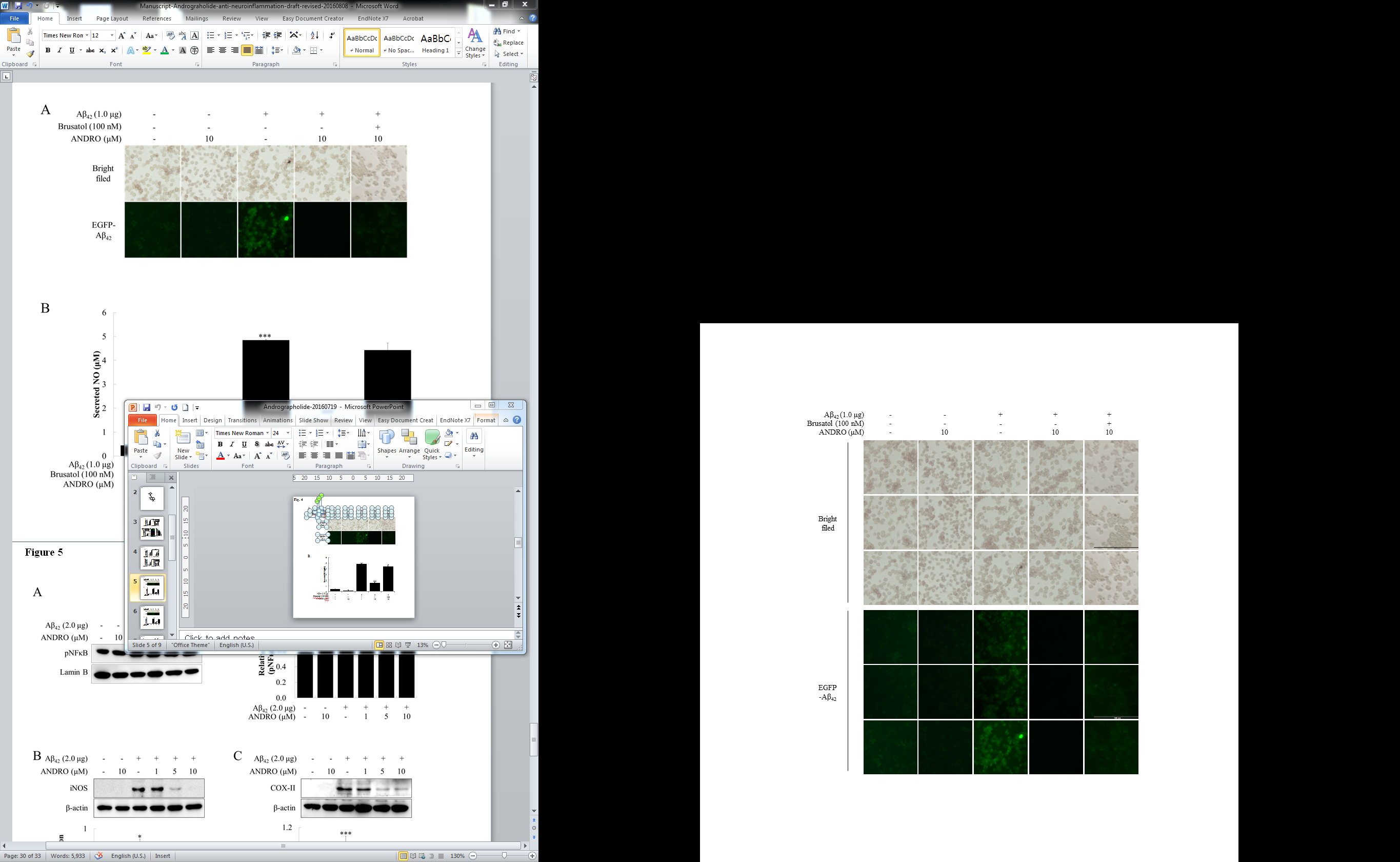
**
